# Supplementary material for: Towards unified AI-driven fracture mechanics: the extended deep energy method (XDEM)
Source: Nat Commun. 2026 Aug 15;17:8492. doi: 10.1038/s41467-026-76748-1 (PMC13484471; doi:10.1038/s41467-026-76748-1)
Supplement: Supplementary file 3 [file 41467_2026_76748_MOESM3_ESM.zip › pic/Results/Stress_intensity_factor/Mode2/different_loads/crack_detailed_fields300.pdf]

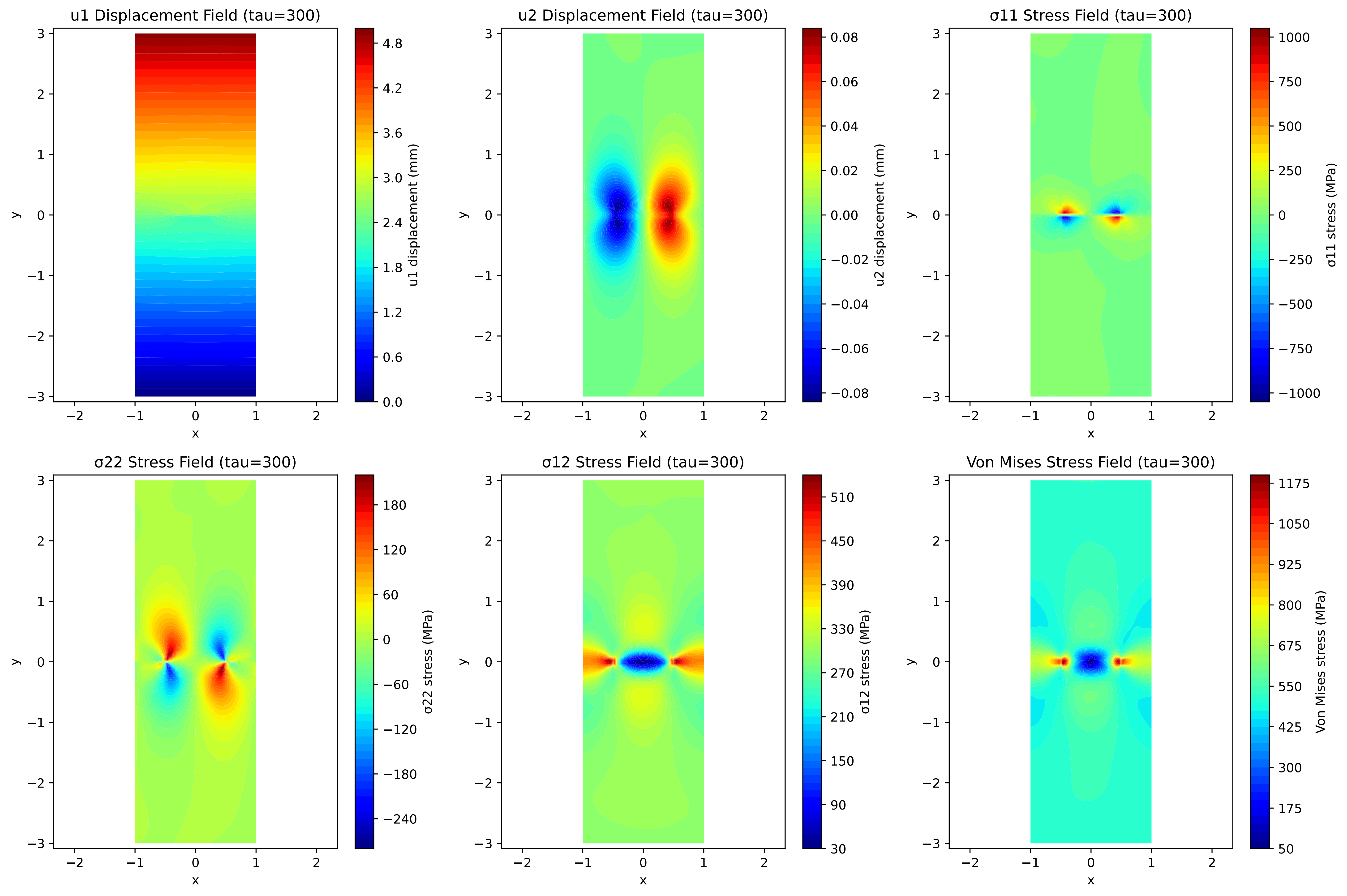
u1 Displacement Field (tau=300)
u1 displacement (mm)
0.0 0.6 1.2 1.8 2.4 3.0 3.6 4.2 4.8
u2 Displacement Field (tau=300)
u2 displacement (mm)
−0.08 −0.06 −0.04 −0.02 0.00 0.02 0.04 0.06 0.08
σ11 Stress Field (tau=300)
σ11 stress (MPa)
−1000 −750 −500 −250 0 250 500 750 1000
σ22 Stress Field (tau=300)
σ22 stress (MPa)
−240 −180 −120 −60 0 60 120 180
σ12 Stress Field (tau=300)
σ12 stress (MPa)
30 90 150 210 270 330 390 450 510
Von Mises Stress Field (tau=300)
Von Mises stress (MPa)
50 175 300 425 550 675 800 925 1050 1175
x
y
−2 −1 0 1 2
−3 −2 −1 0 1 2 3
